# A Confidential QR Code Approach with Higher Information Privacy

**DOI:** 10.3390/e24020284

**Published:** 2022-02-16

**Authors:** Pei-Yu Lin, Wen-Shao Lan, Yi-Hui Chen, Wen-Chuan Wu

**Affiliations:** 1 Department of Electrical Engineering, National Kaohsiung University of Science and Technology, Kaohsiung 807618, Taiwan; pagelin3@gmail.com or; 2 Department of Information Communication, Yuan Ze University, Taoyuan 320315, Taiwan; wenshao0303@gmail.com; 3 Department of Information Management, Chang Gung University, Taoyuan 333323, Taiwan; 4 Kawasaki Disease Center, Kaohsiung Chang Gung Memorial Hospital, Kaohsiung 833401, Taiwan; 5 Department of Computer Science and Information Engineering, National Ilan University, Yilan 260007, Taiwan

**Keywords:** information security, QR code, error correction capability, sensitive information, secret data

## Abstract

In present times, barcode decoders on mobile phones can extract the data content of QR codes. However, this convenience raises concerns about security issues when using QR codes to transmit confidential information, such as e-tickets, coupons, and other private data. Moreover, current secret hiding techniques are unsuitable for QR code applications since QR codes are module-oriented, which is different from the pixel-oriented hiding manner. In this article, we propose an algorithm to conceal confidential information by changing the modules of the QR Code. This new scheme designs the triple module groups based on the concept of the error correction capability. Additionally, this manner can conceal two secret bits by changing only one module, and the amount of hidden confidential information can be twice the original amount. As a result, the ordinary data content (such as URL) can be extracted correctly from the generated QR code by any barcode decoders, which does not affect the readability of scanning. Furthermore, only authorized users with the secret key can further extract the concealed confidential information. This designed scheme can provide secure and reliable applications for the QR system.

## 1. Introduction

Quick Response (QR) codes are widely used in our daily lives since they are convenient and can accommodate a large amount of data [1,2]. Mobile devices can scan the QR code and obtain the data content for public transmission. However, when the information requires privacy for the individual (e.g., e-coupons and e-tickets), current QR Code technologies cannot address the security problems [3,4,5,6]. For example, in 2011, Taiwan High Speed Rail (THSR) released a trial of e-ticketing, in which users could download a QR code after purchasing a ticket via smartphones. These codes replaced paper tickets for entering and exiting the train gate. However, although e-ticketing makes the ride smoother and more environmentally friendly, it also makes it easier for people with bad intentions to forge fake tickets. The security of QR code e-tickets thus remains an unsolved question. Accordingly, it is necessary for companies or e-ticketing systems to hide or protect sensitive QR code content, allowing only authorized users to retrieve it [7].

However, many studies on concealing confidential information have focused on protecting the ownership of digital images or on embedding secrets into digital images [8,9,10,11]. These approaches applied steganography techniques based on traditional media instead of embedding or protecting the QR code directly. Moreover, QR codes have standard encoding and decoding specifications and need to be quickly decoded by scanning devices such as cell phones, tablets, or barcode scanners. In other words, general research on secret image hiding is unsuitable for protecting QR codes.

These schemes [12,13] have explored the security of QR codes, proposing ownership protection via image processing techniques, including the discrete wavelet transform (DWT), the discrete cosine transform (DCT), or the discrete Fourier transform (DFT). These techniques transform QR images into the frequency domain and embed watermarks into the special coefficients of QR images. However, such approaches require computer assistance and are unsuitable for printed QR codes, such as posters or hard paper. In addition, due to the scanning environment of the camera instrument, the generated QR code image may be distorted. Watermarks in the frequency domain may encounter capture problems and cause incorrect watermark results.

Researchers such as [14,15] directly embed the watermark into the barcode by adjusting the height and width of the barcode modules. The module is the black or white square of the barcode. Although such methods directly embed secrets into the barcode modules, the generated barcode still requires additional equipment to calculate the height and width of the modules. In addition, the available barcode scanners cannot directly identify and extract the secrets of the generated QR code, which reduces the practicability.

Different from the schemes presented in [12,13,14,15], Chiang et al. proposed a blind QR code steganographic approach based on the error correction capability [16]. Their method [16] effectively exploits the error correction properties of QR codes and adapts the QR module to embed secrets. A typical scanning device can read the QR data content from the generated QR code. In particular, the authorized recipients can additionally extract secrets from the tagged QR codes. Chiang et al.’s scheme is practical and can be applied to standard mobile devices and scanning devices. However, the maximum payload of the secret is equal to the error tolerance of the QR code. To increase the payload of embeddable secrets, the method proposed by Luo et al. [17] can effectively reduce the modification of the QR module when embedding secrets into QR codes. As a result, the secret payload embedded in the method of Luo et al. is 1.5 times higher than that of the method proposed by Chiang et al.

Based on the above observations, there is a considerable lack of research on protecting the confidential information of QR codes. Anyone can easily scan and retrieve QR data content through barcode readers [16,17,18,19]; therefore, the protection of private data is critical. Traditional image hiding or watermarking techniques require additional image processing processes/computers that are not suitable for real-time scanning and direct retrieval of QR codes by barcode readers. In this article, we aim to design a system to embed secrets into QR codes and to address the following issues: the confidentiality of QR codes and the payload of the confidential information that can be embedded. This proposed method can effectively preserve the original readability of QR codes, significantly reduce the modification of QR modules, and increase the embeddable capacity of secrets in QR codes. Moreover, the embeddable secret payload is superior to related studies [16,17]. Finally, we perform a theoretical analysis to prove the proposed method and discuss its implications.

## 2. The Proposed Confidential QR Approach Based on the Triple Module Group

**Figure 1:**
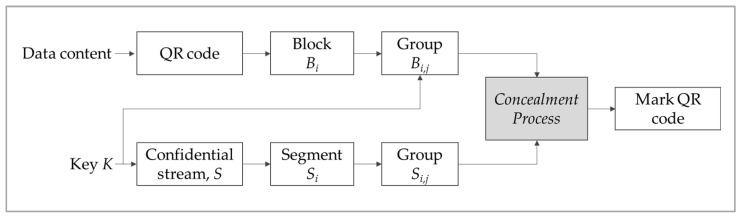
The framework of the proposed QR code approach.

The designed system integrates three QR modules into a group and changes the modules by utilizing the concept of the error correction propriety of QR codes. As a result, the new system can change only one of these modules to hide two confidential information bits for enhancing the confidential capacity. The generated QR code still maintains its virtual QR appearance and readability to reduce tamper risk. Only the authorized user with the secret key can retrieve the hidden secret of the QR code. Figure 1 shows the flowchart of the proposed approach, and below that the algorithm is presented.

### 2.1. Preliminary Process

A QR code is given with data content (such as URL) and the confidential stream. According to QR specification, let *E* be the number of error correction codewords of the QR code and *B_i_* be the *i*-th block of QR code, in which *i* = 1, 2, …, *n*. Based on the error correction propriety of QR codes, this new scheme limits the change amount of QR modules to less than half of *E*. As a codeword consisting of eight modules, we can calculate the maximum change amount of the QR modules *C* as

(1) C=⌊E/2⌋×8.

Let *S* be the confidential stream encrypted with the key *K*. Here, *K* is the secret key of the system dealer. Then the length of *S*, *l_s_*, can be determined as follows:

*l_s_* = 2 × *C*. (2)

As shown in Table 1, QR version 3 and error correction level Q can be taken as an example. Using the number of error correction codewords, *E* = 36, we can learn that *C* = 144 and that the length of the confidential stream *S* is *l_s_* = 288.

To make *S* evenly embedded in the QR code, the proposed system firstly divides *S* into *n* segments, *S_i_*, where *i* = 1, 2, …, *n*. Hence, each segment *S_i_* has *l_s_*/*n* bits. Each segment *S_i_* is subsequently divided into groups, in which each group consists of two bits. Assume that group *S_i,j_* indicates the *j*-th group of the *i*-th segment, for *i* = 1, 2, …, *n* and *j* = 1, 2, …, *C*/*n*. With the key *K*, the system can randomly select 3*C*/*n* modules from the block *B_i_* of the QR code, *i* = 1, 2, …, *n*. The selected modules afterward are divided into groups, in which each group consists of three modules. Assume that group *B_i,j_* is the *j*-th group of the *i*-th block, for *i* = 1, 2, …, *n* and *j* = 1, 2, …, *C*/*n*. *B_i,j_* = 0 means the QR module is white, and *B_i,j_* = 1 means the QR module is black.

Take QR version 3 and error correction level Q. For example, *C* = 144 and *l_s_* = 288. Due to the number of blocks being two (*n* = 2), *S* is divided into segments *S*_1_ and *S*_2_. Each segment has *l_s_*/*n* = 288/2 = 144 bits. The 144 bits of each segment can be paired to derive 72 groups. That is, there are *S*_1,1_, *S*_1,2_, …, *S*_1,72_ for *S*_1_ and *S*_2,1_, *S*_2,2_, …, *S*_2,72_ for *S*_2_. On the other hand, the system with the key *K* can randomly choose 3*C*/*n* = 216 modules from blocks *B*_1_ and *B*_2_ of the QR code. These 216 modules afterward can be divided into 216/3 = 72 groups, that is, *B*_1,1_, *B*_1,2_, …, *B*_1,72_ for *B*_1_ and *B*_2,1_, *B*_2,2_, …, *B*_2,72_ for *B*_2_.

### 2.2. Concealment Process

**Figure 2:**
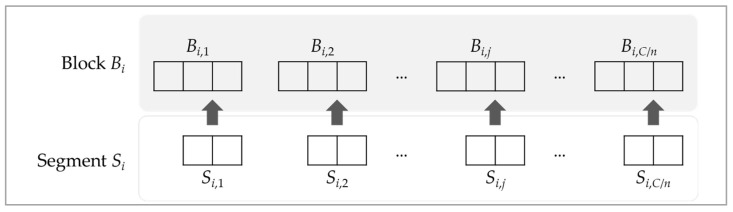
The concealment of segments *S_i_* and the corresponding block *B_i_*.

Figure 2 shows the flowchart of the concealment process. The confidential segment *S_i,j_* can be embedded into the corresponding QR block *B_i,j_* for *i* = 1, 2, …, *n* and *j* = 1, 2, …, *C*/*n.* Let the bits of *S_i,j_* be {*s_2_*, *s_1_*} and the modules of *B_i,j_* be {*b_3_*, *b_2_*, *b_1_*}. The difference value, *d,* can be learned by using *S_i,j_* and *B_i,j_*:

(3) $$d=\sum_{k=1}^{2}b_{k}⊕s_{k}.$$

Here, the notation ⊕ indicates the exclusive-or (XOR) operator. First, consider the value of *b_3_*:
when *b_3_* = 0, adjust a module according to the following formula:
    Case 1: if *d* = 0, unchanged.
    Case 2: if *d* = 1,
          if *b*_1_ = *s*_1_, then set the value of *b*_2_ = *s*_2_; otherwise *b*_1_ = *s*_1_.
    Case 3: if *d* = 2, the *b*_3_ = 1.
when *b_3_* = 1, adjust it according to the following formula:
    Case 4: if *d* = 0, the *b*_3_ = 0.
    Case 5: if *d* = 1,
          if *b*_1_ = *s*_1_, then change the value of *b*_1_ = $\bar{s_{1}}$, otherwise *b*_2_ = $\bar{s_{2}}$.
    Case 6: if *d* = 2, unchanged.

Repeat the above algorithm to process *S_i,j_* and *B_i,j_*, for *i* = 1, 2, …, *n* and *j* = 1, 2, …, *C*/*n*. As a result, the designed system can finally generate the marked QR code with private information. It is worth noting that general users can only obtain the data content (such as URL) on the QR code by using familiar barcode readers but will not know the private information hidden in it. Furthermore, QR codes are meaningless images to the human eye. Thus, changing the QR modules does not cause any suspicion to users. Due to the proposed approach modifying the modules within the limit of the error correction capability, the readability of the generated marked QR code can be guaranteed.

### 2.3. Extraction Process

To retrieve the confidential stream, the authorized users with the key *K* are allowed to further reveal the *S* from the generated QR code in the following way. According to the QR version and the error correction level, the extraction process can learn the number of error correction codewords *E*, the number of error correction blocks *n*, and the values of *C* and *l_s_* by Equations. (1) and (2).

Firstly, the system can extract the assigned 3*C*/*n* modules for each QR block, *B_i_*, by using the key *K*, where *i* = 1, 2, …, *n*. The 3*C*/*n* modules, afterward, are divided into groups by gathering three modules into one group. Thereby, there are *C*/*n* groups for each block *B_i_*. Let *B_i,j_* be the *j*-th group of the *i*-th block, for *i* = 1, 2, …, *n* and *j* = 1, 2, …, *C*/*n*. *B_i,j_* = 0 means the QR module is white, and *B_i,j_* = 1 means the QR module is black.

Assuming {*b_3_*, *b_2_*, *b_1_*} are the QR modules of group *B_i,j_*, one can extract the corresponding confidential bits {*s_2_*, *s_1_*} with the following equation:

(4) $$\mathrm{if}\;b_{3}=0,\;\mathrm{then}\;s_{1}=b_{1},\;s_{2}=b_{2}\;\mathrm{else}\;s_{1}=\bar{b_{1}},\;s_{2}=\bar{b_{2}}.$$

The results {*s_2_*, *s_1_*} are the confidential groups corresponding to *S_i,j_*. The group *S_i,j_* indicates the *j*-th group of the *i*-th segment, for *i* = 1, 2, …, *n* and *j* = 1, 2, …, *C*/*n*. Repeat all the steps according to the above decoding method and extract all {*s_2_*, *s_1_*} from *B_i,j_*, for *i* = 1, 2, …, *n*, and *j* = 1, 2, …, *C*/*n*. Finally, the authorized users with the key *K* can decrypt *S* and obtain the confidential stream.

## 3. Experimental Results

The designed concealment QR system can embed confidential information and retain the readability of the generated QR code. In particular, the proposed algorithm takes three modules as a group, and only one module being changed can help hide two bits of confidential information. Therefore, this method can effectively reduce the adjustment to the QR modules, enhance the embedded sensitive information, and retain the error correction capability of the QR code. In the following, the essentials of the confidential capacity, the probability of modified QR modules, and the readability of the generated marked QR code are discussed.

### 3.1. Capacity

Table 2 shows the payload of the confidential stream under QR code versions 1, 10, 20, 30, and 40. The larger the QR version is, the more QR size and data content there are. There are four error correction levels, L, M, Q, and H, for all QR versions. With a larger level, the value of *C* is higher as defined in Equation (1). For instance, in QR version 10, *C* is 288 modules for error correction level L, and *C* is 896 modules for error correction level H. 

The designed triple module group system can enhance the maximum amount of confidential information to *l_s_* = 2 × *C*. The last column of Table 2 lists the capacity of confidential information *l_s_* under different QR versions. Since the proposed approach can reduce the changes to the QR modules, it can significantly increase the amount of embeddable confidential information. Furthermore, it can be observed that increasing the QR version and error correction level can help embed more confidential stream *l_s_* (bits). In QR version 10, the embeddable payload *l_s_* = 576 bits when the error correction level is L, and *l_s_* = 1792 bits when the error correction level is H. Therefore, the developers can embed more confidential information by increasing the QR code version and error correction level.

### 3.2. Modification

The notation *Ϻ* in Table 3 indicates the total number of QR modules (except the function pattern modules, the format, and the version information modules). The value of *C*/*Ϻ* denotes the percentage of maximum changeable modules for QR codes. We can note that the rates are around 10%, 19%, 28%, and 32% for error correction levels L, M, Q, and H, respectively. The modification ratio *γ* in Table 3 represents the average rate of the changed modules of our generated QR code. We can observe that the values of *γ* are lower than that of *C*/*Ϻ* for different QR versions and error correction levels. Here, the theoretical value *γ* can be calculated as:

(5) $$\gamma =\frac{2}{3}C\;(\%)$$

In the concealment process, each group *B_i,j_* will meet one of the conditions from case 1 to case 6. Considering cases 2 to 5, the probability of each group being changed is 2/3. The other 1/3 chance is that the original modules remain unchanged in case 1 and case 6. Therefore, the modification ratio *γ* of the proposed approach can be derived as 2/3*C*. 

### 3.3. Readability

Based on the error correction level of QR code specification, if the damaged module is under *C*/*Ϻ* module changes, the original QR data content can be recovered correctly. As the results in Table 3 demonstrate, the *γ* values of the designed method are less than the values of *C*/*Ϻ* under all QR versions and error correction levels. Therefore, it can guarantee that one can correctly decode and reveal the original QR data content (such as URL). Moreover, the readability of the generated marked QR code can reduce the risk of suspicion. Furthermore, the receivers with the key *K* are authorized to extract the confidential stream of the marked QR code.

### 3.4. Comparison

The schemes [12,13] transform the QR image into the frequency domain and requires computer assistance. Additionally, the watermark in the frequency domain may encounter capture problems, resulting in incorrect watermark results. Methods [14,15] use the adjustment of the module’s height and width to embed the watermark. Their generated barcodes require additional equipment to calculate the module’s height and width differences. Moreover, existing barcode scanners cannot directly identify and extract the secrets of their generated barcodes, thus reducing practicality [12,13,14,15]. 

In contrast, the schemes [16,17] and our approach conceal the secrets in QR codes by adjusting the QR modules directly. The computational complexity of the methods [16,17] and our system is lower than the related works [12,13,14,15]. Our designed concealment and extraction operations are based on the QR code specifications and can be utilized on mobile applications or scanning devices without additional equipment. A typical scanning device can read the QR modules directly from the generated QR code.

**Figure 3:**
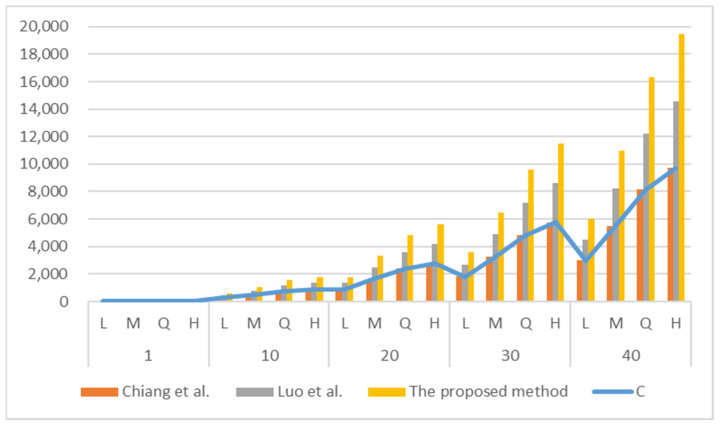
The comparisons of the embeddable payload of confidential stream between the proposed approach and the related schemes.

Figure 3 demonstrates the proposed system with the related QR module modification schemes regarding the embeddable capacity of the confidential secrets. The X-axis represents the four error correction levels under different QR versions, and the Y-axis represents the confidential stream (bits) that can be carried. The payload of the confidential stream of Chiang et al.’s method [16] is equal to the maximum changeable QR modules *C* (as in Equation (2)). For the same QR version, Luo et al.’s scheme [17] can embed more payloads of the confidential stream into the QR code. The embedding capacity of the method presented in [17] is 3/2×*C*, i.e., 1.5 times that of the method presented in [16]. 

Our designed triple module group approach can effectively reduce the alteration to the QR modules. Therefore, the proposed method can enhance the embedding capacity of the confidential information to *l_s_* = 2 × *C*. Figure 3 shows that the embeddable confidential stream of our proposed system is higher than methods [16,17]. In addition, the new system can effectively hide more secrets with larger QR versions and higher error correction levels.

Only an authorized recipient with the key *K* can further decode and achieve the confidential streams *S* correctly in terms of security. However, the intruder may use the brute-force attack to infer the contents of *S* without knowing the key *K*. We used the brute-force attack to evaluate the security of the related QR methods [16,17] and the proposed system. Table 4 lists the computations required for the brute-force attack in different QR versions and error correction levels. Since the confidential payloads *l_s_* of methods [16,17] are *C* and 1.5*C*, respectively, the cracking probability *ρ* is 2^−*C*^ and 2^−1.5*C*^. On the other hand, the *l_s_* of our proposed method is 2*C*, and the cracking probability *ρ* is 2^−2*C*^. Accordingly, the security of the proposed method is higher than that of the methods presented in [16,17] in the brute-force attack. Furthermore, as the QR code version and the error correction level increase, the security also increases.

According to the results, the designed approach is feasible for embedding a confidential stream into QR codes. The generated QR code can satisfy the requirements of reducing the amount of the modification of QR modules, thus increasing the capacity of the embeddable confidential information, maintaining the readability of the QR data code, and authorizing the users to decrypt and retrieve the confidential information with *K*.

## 4. Conclusions

Conveying the confidential information of QR codes has been a significant issue recently. Many mobile devices apply QR codes for mobile payments, e-tickets, e-bonuses, and digital signatures. As a result, the private information needs to be protected from casual scanning. The designed concealment process is based on the error correction capability of QR codes, which can preserve the readability of the generated marked QR code. General users can scan to bring the QR data content to avoid being suspected and maliciously attacked by others. Only authorized users with the secret key can further extract the confidential stream with scanners or mobile applications.

The designed triple module grouping approach conceals two confidential bits by changing only one QR module. Accordingly, the new approach can effectively reduce the alteration of the QR modules and improve the embeddable confidential capacity of a QR code. The confidential payload of the new system is twice the error correction capability of a QR code, which can solve the issue of insufficient private payload, and the performance is better than the related schemes. Moreover, the decoder and extraction operations of the proposed system are based on the QR specification. A usual scanning device can read the QR modules (including the QR data content and the encrypted secret *S*) directly from the generated QR code. Therefore, the extraction of *S* is the same as the original QR operation. However, the new scheme needs to decrypt *S*. Encryption and decryption can be determined according to system requirements. Here, we can utilize a one-way hash function for encryption and decryption to reduce the time complexity. Therefore, the computational complexity of the designed system is low and can be widely applied on mobile applications or scanning devices. The algorithm can also be practical for various two-dimensional barcodes with error correction capabilities.

## Figures and Tables

**Table 1:** The error correction characteristics for QR code version 3.

Error Correction Level	Number of Error Correction Blocks (*n*)	Total Number of Codewords	Number of Data Codewords	Number of Error Correction Codewords (*E*)
L	1	70	55	15
M	1	70	44	26
Q	2	70	34	36
H	2	70	26	44

**Table 2:** The capacity of the confidential stream under different QR code versions.

Version	Error Correction Level	*C*	Confidential Capacity, *l*_s_ (bit)
1	L	24	48
	M	40	80
	Q	48	96
	H	64	128
10	L	288	576
	M	520	1040
	Q	768	1536
	H	896	1792
20	L	896	1792
	M	1664	3328
	Q	2400	4800
	H	2800	5600
30	L	1800	3600
	M	3248	6496
	Q	4800	9600
	H	5760	11,520
40	L	3000	6000
	M	5488	10,976
	Q	8160	16,320
	H	9720	19,440

**Table 3:** The average change rates of QR code versions 1, 10, 20, 30, and 40.

Version	QR Modules, *Ϻ*	Error Correction Level	*C*/*Ϻ* (%)	Modification Ratio, *γ* (%)
1	208	L	12%	8%
		M	19%	13%
		Q	23%	15%
		H	31%	21%
10	2768	L	10%	7%
		M	19%	13%
		Q	28%	18%
		H	32%	22%
20	8683	L	10%	7%
		M	19%	13%
		Q	28%	18%
		H	32%	21%
30	17,483	L	10%	7%
		M	19%	12%
		Q	27%	18%
		H	33%	22%
40	29,648	L	10%	7%
		M	19%	12%
		Q	28%	18%
		H	33%	22%

**Table 4:** The amount of computation required for the brute-force attack under different QR versions.

Version	Error correction level	Cracking Probability *ρ*		
		[16]	[17]	Ours
1	L	2^−24^	2^−36^	2^−48^
	M	2^−40^	2^−60^	2^−80^
	Q	2^−48^	2^−72^	2^−96^
	H	2^−64^	2^−96^	2^−128^
10	L	2^−288^	2^−432^	2^−576^
	M	2^−520^	2^−780^	2^−1040^
	Q	2^−768^	2^−1152^	2^−1536^
	H	2^−896^	2^−1344^	2^−1792^
20	L	2^−896^	2^−1344^	2^−1792^
	M	2^−1664^	2^−2496^	2^−3328^
	Q	2^−2400^	2^−3600^	2^−4800^
	H	2^−2800^	2^−4200^	2^−5600^
30	L	2^−1800^	2^−2700^	2^−3600^
	M	2^−3248^	2^−4872^	2^−6496^
	Q	2^−4800^	2^−6700^	2^−9600^
	H	2^−5760^	2^−8640^	2^−11,520^
40	L	2^−3000^	2^−4500^	2^−6000^
	M	2^−5488^	2^−8232^	2^−10,976^
	Q	2^−8160^	2^−12,240^	2^−16,320^
	H	2^−9720^	2^−14,580^	2^−19,440^
